# A Dynamic 3D Tumor Spheroid Chip Enables More Accurate Nanomedicine Uptake Evaluation

**DOI:** 10.1002/advs.201901462

**Published:** 2019-10-04

**Authors:** Jialang Zhuang, Jie Zhang, Minhao Wu, Yuanqing Zhang

**Affiliations:** ^1^ Guangdong Key Laboratory of Chiral Molecule and Drug Discovery School of Pharmaceutical Sciences Sun Yat‐sen University Guangzhou Guangdong 510006 P. R. China; ^2^ Department of Immunology Zhongshan School of Medicine Sun Yat‐sen University 74 Zhongshan 2nd Road Guangzhou 510080 P. R. China

**Keywords:** 3D culture, cellular uptake, microfluidics, mesoporous silica particles (MSNs), nanomaterials

## Abstract

Nanomedicine has brought great advances for drug delivery by improving the safety and efficacy of pharmaceuticals. However, many nanomaterials showing good distribution property in vitro often display poor cellular uptake during in vivo administration. Current cellular uptake research models are mainly based on the traditional 2D culture system, which is a single layer and static system, thus the results cannot accurately reflect the distribution of nanoparticles (NPs) in vivo. In the present study, a multiple tumor culture chip (MTC‐chip) is constructed to mimic solid tumor and dynamic fluid transport, in order to better study NPs penetration in vitro. Cellular uptake of mesoporous silica particles (MSNs) is evaluated using the 3D tumor spheroids on chip, and it is found that: 1) continuous administration results in larger MSNs penetration than transient administration at the same dose; 2) the size effect on cellular uptake is less significant than reported by previous in vitro studies; and 3) pretreatment with hyaluronidase (HAase) enhances the tumor penetration of large‐size MSNs.

## Graphical abstract

The multiple tumor culture chip is used to more accurately study mesoporous silica particles (MSNs) uptake and it is found that 1) continuous administration results in larger MSNs penetration than transient administration; 2) the size effect on cellular uptake is less significant than reported by previous in vitro studies; and 3) pretreatment with hyaluronidase (HAase) enhances the tumor penetration of large‐size MSNs.

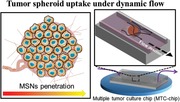

## 1 Introduction

The rapid development of nanoparticles (NPs) significantly improves the tumor therapy of drug transport by providing protection from degradation and enhanced permeability and retention effect.^1^ Although nanomedicine displays great therapeutic potential in the laboratory and preclinical research, only limited therapeutic benefits have been achieved in clinical trials.^2^, ^3^ Nowadays, differences in cell uptake efficiency and toxicity of NPs between in vitro and in vivo studies remain huge because the traditional cell model cannot completely replicate the physiological environments in the body. Besides, many reports have shown that the cellular uptake of NPs largely depends on their physicochemical characteristics.^4^, ^5^, ^6^, ^7^ Since the above conclusions are mainly derived from in vitro experiments based on common 2D evaluation models, which might not be fully reliable because of the absence of the complex microenvironment such as fluid flow, vasculature, extracellular matrix (ECM) and so on, fully following such conclusions to develop NPs might lead us to miss some of the promising nanocarriers.^8^, ^9^

**Figure 1:**
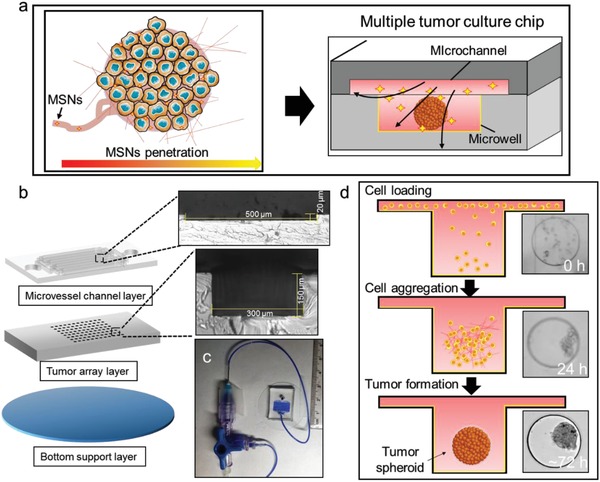
MTC‐chip design and protocol. a) MTC‐chip for NPs penetration study. b) Schematic view and optical microscopic images of microfluidic device with three layers. The depth of the microchannel is approximately 20 µm while the depth of the microwell is about 150 µm. c) Photograph of the whole device. d) Workflow of the 3D culture on chip.

To date, most of the NPs cellular uptake evaluation models are based on 2D monoculture models, in which cells were statically incubated in the media containing a constant concentration of NPs. Theoretically, the results from such experiments might be reliable if NPs can well spread in the body as small molecule did. In fact, some NPs can easily aggregate or sediment in such systems, increasing the concentration of NPs on the surface of adhering cells.^10^, ^11^ Moreover, these platforms only allow researchers to recapitulate several aspects of the tumor microenvironment (TME) for NPs study. Structural components of TME including the ECM and tumor interstitial fluid contribute to the asymmetrical distribution of NPs within tumors but remain absent in most traditional models.^12^, ^13^ Current 3D multicellular tumor spheroid (3D‐MCTS) models could indirectly apply natural or synthetic support material to provide basic ECM and tumor scaffolds for cell culture.^14^ However, these studies remain to be static models, which cannot reflect the penetration properties of NPs under dynamic fluid flow systems. Thus, a liquid flow system mimicking the interstitial flow in cancer is also required to integrate into the 3D tumor evaluation system. Recently, microscale channels can successfully conduct fluidic flows to build the microvessels‐like structures in the microfluidic device.^15^, ^16^ However, the microchannels including its accessories could generate the shear stress that causes cell damage and changes the uptake of nanomaterials.^17^, ^18^ To address this issue, the reduction of shear stress in microfluidic devices needs to take into consideration. In the present paper, we fabricated a multiple tumor culture chip (MTC‐chip) to address the problems mentioned above. We produced a bulk of 3D tumor spheroids and incorporated a dynamic administration system in the MTC‐chip, to assess the cellular uptake of mesoporous silica nanoparticles (MSNs) in breast cancer MCF‐7 spheroids under indirect shear force. Recently, MSNs have been served as a multifunctional nano‐delivery system for cancer therapy, and their biodegradability, as well as compatibility, has been proved in animal studies.^19^ We demonstrate the capability of MTC‐chip to investigate MSNs penetration, and found that 1) continuous administration was better than transient administration for MSNs penetration at the same dose; 2) size effect on cellular uptake within the spheroids in 3D dynamic condition was smaller than that in 2D static model; 3) HAase enhanced the large‐size MSNs penetration in tumor spheroids. In summary, we tried to build a relevant model for such tissue as solid tumors surrounded by a dense stroma of matrix and a dynamic interstitial fluid flow, to study the MSNs delivery on chip, which provides an alternative method for MSNs cellular uptake prediction (**Figure** **1**a).

## 2 Results and Discussion

### 2.1 Microfluidic Chip Design and Operation

In the present study, based on the idea of “solid tumor embedded with a dynamic interstitial fluid network” and the purpose of MSNs penetration study, MTC‐chip needed the requirements as follow: 1) 3D tumor cell culture, 2) transport interstitial fluid, 3) liquid sampling with an active concentration control, 4) microscopic observation of the whole chip with bright field and fluorescence imaging. Inspired by the design of the independent 3D culture chamber based on the previous studies,^20^, ^21^ a multiple tumor array layer (Figure 1b,c), which was composed of 256 microwells (diameter × height = 300 × 150 µm), was used for spheroids formation in MTC‐chip. On this basis, we added the microchannel layer that could realize the microenvironment system of dynamic liquid flow in MTC‐chip. The microchannel layer was composed of 16 microchannels (length × width × height = 7500 × 500 × 20 µm), which was built on the multiple tumor array layer. At last, a glass slide was used as a supporting substrate. In detail, the microchannels in the top layer were used to supply fluid flow and mimic dynamic interstitial fluid transport while the microwells in the middle layer were used for 3D‐MCTS formation. The device was fabricated by a modified soft lithography method (Figure S1, Supporting Information). The fluid simulation results (Figure S2, Supporting Information) demonstrated that part of the fluid would fall to the microwells while the linear velocity gradually decreased to 0.01 mm s^−1^, therefore some of the cells from cell suspension will stagnate at the bottom of the microwells during cell loading (Figure 1d). Moreover, MTC‐chip was pretreated with Pluronic F‐127 (1%) to block cell adherence before cell loading,^22^ which is essential to transform 2D culture into 3D culture. Then, a three‐way mixing valve was placed at the inlet of the device, which could be used to introduce up to two different solutions into the microchannels. Depended on the special inlet, we can achieve different routes of drug administration, such as intravenous (IV) infusion and IV bolus, with two independent syringe pumps. Moreover, since cells in the microwells were indirectly exposed to the flow medium during the sample loading, which could reduce the shear force of the fluidic conducted by the device.

### 2.2 Microfluidic Cell Culture

**Figure 2:**
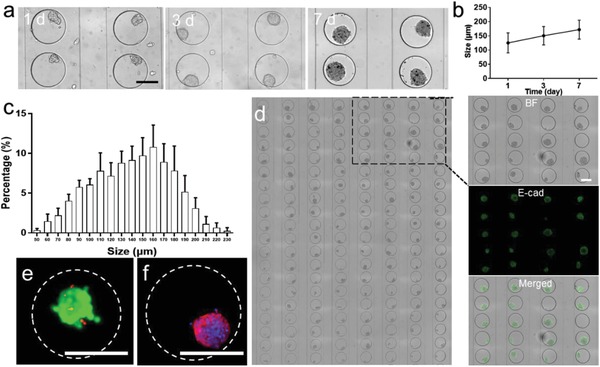
Investigation of 3D culture on the chip. a) Optical images and size of MCF‐7 spheroids after 1, 3, and 7 days culture, scale bar, 200 µm. b) Growth of 3D tumors size on chip. c) Size distribution of the 3D tumors on chip. d) Bright field images of 160 MCF‐7 tumors on chip and image analysis on 20 microwells filled with spheroids stained for E‐cadherin, scale bar, 200 µm. e) Fluorescent image of a tumor stained by Calcein‐AM/PI visualizing live/dead (green/red) cells, scale bar, 200 µm. f) Fluorescent image of F‐actin (red) and nuclear (blue) staining for cytoskeletal presentation, scale bar, 200 µm.

Next MCF‐7 cells were introduced into the microchannel from the outlet at different flow rates and loading time to maximize the cell loading amount in each microwell. After cell loading, we found each microwell contained about 120 cells (flow rate for 2 µL min^−1^ and loading time for 10 min) (Figure S3, Supporting Information). Once the cells were seeded, the device was placed in an incubator at 37 °C, 5% CO_2_, and 100% humidity for up to 7 days while the culture medium was replaced by a gentle microfluidic flow for 15 min every 2 days. Loose tumor spheroids were first established after 3 days of cell culture on chip. To construct the tumor stroma in 3D culture, cells were co‐cultured with matrigel to generate tumor spheroids in MTC‐chip^23^ (Figure S4, Supporting Information). After cell seeding, cells began aggregating within 24 hours and formed compact spheroids after 3 days (Figure S5, Supporting Information). The size of tumor spheroids gradually increased on chip during a 7‐day culture (**Figure** **2**a,b). Our microfluidic platform could generate about 200 3D tumor spheroid s (≈150 µm) after 7 days. Immunofluorescence staining was also applied to evaluate the tumor spheroids on chip. It is noticeable that E‐cadherin (E‐CAD), which can maintain MCF‐7 cell–cell junctions, was found to be a high level in these 3D tumors (Figure 2c).^24^ Furthermore, the expression of collagen IV was detected (Figure S6, Supporting Information), which indicated the presence of ECM in the spheroids. Meanwhile, the survival rate of MCF‐7 within spheroids after 7 days was 88.1%, while morphology analysis revealed that 3D‐MCTS on chip obtained a mature F‐actin cytoskeleton. The above results confirmed the high viability and integrity of the spheroids produced by the microfluidic system (Figure 2d,e). Moreover, the impact of cell density on cell loading was determined (Figure S7, Supporting Information). Although a higher amount of seeded cells could lead to lager tumor spheroid in principle, we found that cells cannot get enough nutrients and gas supply in the case of 2 × 10^7^ cells mL^−1^, which might fail to form compact tumor spheroid. These results demonstrated that our platform enables multiple tumor spheroids production, which indicated the feasibility of further investigation of the NPs uptake assay or drug delivery test in the 3D cancer model by this method.

### 2.3 Effect of the Route of Administration on NPs Penetration

**Figure 3:**
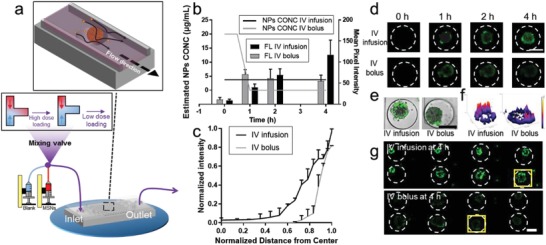
Characterization of MSN_90_ penetration of MCF‐7 spheroids by continuous administration (IV infusion) and transient administration (IV bolus). a) Perspective view of applying different routes of MSNs administration on chip with three‐way mixing valve inlet. b) Estimated concentration (CONC) of MSN_90_ in the microchannels on chip and the particokinetics of MSNs within the spheroids by IV infusion or IV bolus. c) Normalized fluorescent intensity distribution of NPs as a function of distance from the center of the spheroid. d) Time lapse images of MSN_90_ penetration by IV infusion or IV bolus in one 3D tumor during administration. e) Merged images of MSN_90_ penetration by IV infusion or IV bolus in one 3D tumor, scale bar, 200 µm. f) Surface plot images of one MCF‐7 (based on the fluorescent images of (g)). g) Cellular uptake of MSN_90_ after different routes of administration in eight tumor spheroids, scale bar, 100 µm.

Compared with the nonmicrofluidic 3D cell culture system, one advantage of the MTC‐chip is that the inlet can be connected to multiple reservoirs to adjust the drug concentration arbitrarily. So this device can be used to study multiple routes of administration. We chose two administration models to test the chip, including continuous administration such as IV infusion and transient administration such as IV bolus. According to the structural characteristics of MTC‐chip and the fluid characteristics of perfusion (the viscosity was about 0.94 Cp and the density was about 1 g mL^−1^), we selected a perfusion flow rate of 15 µL min^−1^. Laminar flow occurred in the microchannels during the NPs administration. But the laminar flow would become unsteady when fluid moved above the microwells. The shear force of the fluid was 4.418 dyn cm^−2^ at this flow rate, thus the result was close to the average shear force of normal microvascular tissue (4 dyn cm^−2^). Meanwhile, the microwells were designed to protect the cells from high shear stresses because the fluid only applied an indirect impact on tumor spheroids on chip. In this study, MSNs with diameters about 90 nm were applied to interact with 3D tumors in the microwells. Considering different administrations could result in different MSNs levels in circulating system, the concentration of MSNs in the microchannel was organized by adjusting the flow rate of pump A (MSNs solution) and pump B (blank medium) (**Figure** **3**a). Thus, theoretically MSNs level could be maintained at a steady low concentration during continuous administration while MSNs concentration began with a high level and then decreased during transient administration. Moreover, both of these two administrations shared the same dose during the experiment. The fluorescent intensity of MSNs was quantified based on the captured images during the whole experiment (Figure S8, Supporting Information). The accumulation in 3D tumors after transient administration tended to decrease when MSNs level dropped back to zero (Figure 3b). Meanwhile, MSNs penetration by continuous administration was higher than that after transient administration (Figure 3c). Detail information on the surface plots and normalized fluorescence intensity distribution of MSNs confirmed that continuous administration could result in deeper and stronger penetration in the spheroids (Figure 3d–f). Transient administration was less associated with MSNs' retention in tumor spheroids while continuous administration could be favorable for MSNs' accumulation. The route of administration also contributed to the distribution profile of MSNs and influenced the accumulation. For instance, the intensity associated with continuous administration was found to be significantly higher within 3D tumors, whereas most of the signals obtained by transient administration were represented at the edge of the spheroids. This study was also aimed at selecting the best administration method for MSNs delivery for cancer therapy. Together, the above results demonstrated continuous administration might be a better administration method for MSNs with larger NPs accumulation.

### 2.4 Effect of the Size of MSNs on NPs Penetration after a Single Dose Continuous Administration NPs Delivery

**Figure 4:**
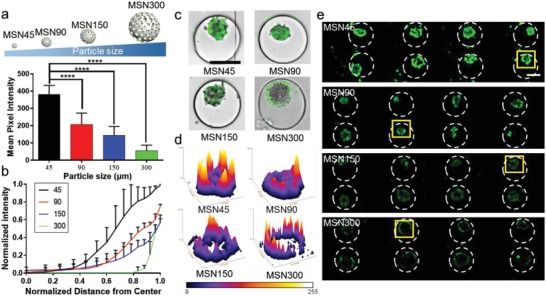
Characterization of MSNs penetration of MCF‐7 spheroids by MSNs with different size. a) Mean fluorescence intensity of cells after the internalization of MSNs. b) Normalized fluorescent intensity distribution of NPs as a function of distance from the center of the spheroid after MSNs loading. c) Merged images of penetration of MSNs in one 3D tumor, scale bar, 200 µm. d) Surface plot images of one MCF‐7 spheroid (based on the fluorescent images of (e)). e) Cellular uptake of MSNs in eight tumor spheroids, scale bar, 100 µm.

Another advantage of this chip is the ability to study the uptake of NPs under weak shear stress in a nonstatic microenvironment, which is very important for nanocarriers of different sizes. We investigated many previous reports on the size effect of MSN in cellular uptake,^25^, ^26^ and found that most of them were carried as traditional cellular assays under static condition. Our above results indicated that the administration route can influence the NPs penetration, which indicated the effect of fluid flow on cellular uptake. Thus, we intended to explore the size effect of MSNs on NPs penetration under dynamic administration. Four types of MSNs with increasing diameters were synthesized and abbreviated as MSN_45_ to MSN_300_ (Figure S9 and Table S1, Supporting Information) in the present study. Our findings indicated that the size effect on cellular uptake of MSNs was applied to MSNs penetration during *continuous* administration (**Figure** **4**a,b). Specifically, small‐size MSNs showed a stronger tendency to be accumulated in the tumor spheroids and retain there at a higher level compared to large‐size MSNs (300 nm). Moreover, we found that smaller particles could be closer to the center within 4 h while large‐size MSNs with larger particle size cannot allow effective diffusion into 3D tumors (Figure 4c–e). The results of MSNs distribution in spheroids showed that cellular uptake of large‐size MSNs was one‐quarter of that for small‐size MSNs (90 nm). It is worth noting that it was almost impossible for cells to uptake any large size MSNs (Figures S10 and S11, Supporting Information) in 2D cell culture and spheroids under static incubation, which was constant with previous in vitro studies.^27^, ^28^ It seems that such traditional static models might amplify the size effect on cellular uptake. Moreover, recent in vivo models^29^, ^30^ have shown that the size effect on cellular uptake was not as severe as in those in available literature in vitro reports, those large‐size MSNs could be distributed in target tissue although small MSNs could be better absorbed. Similar results were obtained by MTC‐chip, as other described in vivo researches did.^31^, ^32^ The above findings indicated that maybe some promising NPs, which could be potentially absorbed in the body and reach the target organ in animal models, have failed to pass such previous static in vitro experiments and were excluded from further analysis. Thus, we suggested that some NPs may need to be re‐evaluated because of the poor evaluation ability of such static models.

### 2.5 Effect of ECM Pretreatment of Tumor Spheroids on MSN_300_ Accumulation

**Figure 5:**
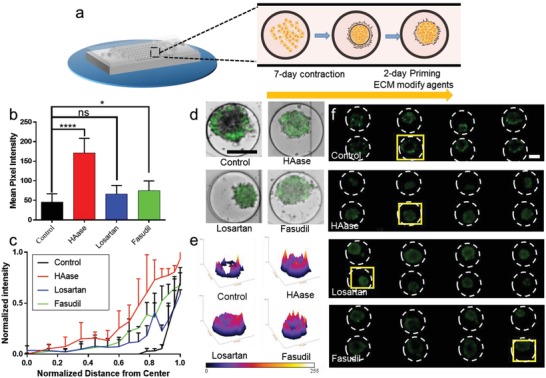
Characterization of MSN_300_ penetration of MCF‐7 spheroids by different ECM pretreatment. a) Schematic of the experimental design. After 3D tumor formation, MCF‐7 spheroids were treated with different ECM modified agents for 2 days before MSNs loading. b) Mean fluorescence intensity of cells after the internalization of MSNs. c) Normalized fluorescent intensity distribution of NPs as a function of distance from the center of the spheroid after MSNs loading. d) Merged images of penetration of MSNs in one 3D tumor, scale bar, 200 µm. e) Surface plot images of one MCF‐7 spheroid (based on the fluorescent images of (f)). f) Cellular uptake of MSNs in eight tumor spheroids, scale bar, 100 µm.

Although nanocarriers with larger diameters indicate poorer permeability and lower cell uptake rates, higher drug loadings and better stability make researchers reluctant to abandon these large‐size nanocarriers. Nowadays various approaches such as NPs' surface conjugation have been applied to enhance tumor penetration of these large‐size NPs.^33^ Among these strategies, co‐administration with matrix modifiers, which can modulate the tumor microenvironment via pharmacological efforts, has been widely applied to enhance tumor penetration in clinical studies. Among them, some drugs can improve the uptake of NPs by regulating the tumor microenvironment. Our microfluidic devices could construct 3D tumors and tumor microenvironment in a certain extent, which could be used to test such pharmacological modulations. In this study, three therapeutic drugs that could reduce ECM were used to test whether the uptake and distribution of MSN_300_ in the tumor spheroids was improved. 3D tumors were treated with candidate compounds for 2 days before MSN_300_ administration (**Figure** **5**a). Our results indicated that these three matrix modifiers can promote MSN_300_ penetration in varying degrees. Pretreatment with hyaluronidase (HAase), which could be served as an ECM‐degrading enzyme and degrade hydrogels,^34^ significantly enhanced the effective tumor penetration of MSN_300_. Both of the cellular accumulation of fluorescence intensity and the deeper biodistribution of NPs were detected in HAase treated group (Figure 5b,c). Previous findings indicated that Losartan could be used to enhance the accumulation of intravenously administered NPs such as Doxil via inhibiting angiotensin‐II‐receptor 1.^35^, ^36^ However, our observations revealed that most of MSN_300_ still appeared at the edge of the spheroids in the Losartan pretreated group (Figure 5d–f). Although ROCK inhibitor Fasudil can disrupt ECM integrity by decreasing the contraction of tumor spheroids,^37^ the distribution of MSNs could not be benefited from the effect of Fasudil. Moreover, a deeper tumor penetration of HAase group in the tumor center was observed during the experiment. In summary, HAase pretreatment resulted in more MSNs penetration when compared with other groups. Since MTC‐chip could not fully mimic the complex microenvironment system as the animal did, the promotions of NPs penetration by such treatments were not reflected in MTC‐chip. We assumed that HAase contributed a direct impact on ECM, which can be more effective in MTC‐chip, while Losartan and Fasudil could only present indirect effects on ECM. Together, the above results demonstrate the potential of our device to test pretreatment strategies for improving the distribution of nanomedicine.

## 3 Conclusions

In conclusion, a high‐throughput, biomimetic, and dynamic administration system has been presented, which enables the production of a number of MCF‐7 spheroids on chip. 3D‐MCTS, ECM, and interstitial fluid systems were successfully integrated into the microfluidic platform, which can offer a physiological condition closer to TME for the exploration of NPs penetration. The present quantitative analysis of the MSNs penetration in 256 individual spheroids on MTC‐chip provides pieces of evidences on the cellular uptake of MSNs in the early state of nanomedicine discovery. Moreover, this platform could also be used for the culture of primary liver cancer‐derived organoids after raising the height of the microchannels (Figure S12, Supporting Information). Such an approach provides a microfluidic‐based method for the study of NPs in mimic organ‐level. Meanwhile, the MTC‐chip allowed the simultaneous screening of matrix modifiers by extending to a microarray with 16 inlets or more, which might reduce the batch effect of the device and raise the accuracy of the system. Previous studies have efficiently achieved 3D culture by microfluidic technology, but little of them have accessed the NPs penetration in such system, let alone investigate the cellular uptake of NPs under dynamic administration as this work did. Moreover, the microdevice could be upgraded by the integration of various stromal cells in further study. Meanwhile, it allowed for the real‐time monitoring of penetration of MSNs within spheroids on chip. We demonstrated that the route of administration can determine MSNs penetration, and continuous administration of nanomedicine resulted in larger NPs accumulation in the spheroid than transient administration did on MTC‐chip. Our results also revealed that size effect on cellular uptake of MSNs could be also applied in 3D flow condition. However, the traditional static incubation system was assumed to be exaggerated the role of MSNs size on cellular uptake. These studies might produce misleading data and let us miss those potential NPs for further research. HAase, which can directly degrade tumor collagen content, enhanced diffusion of large‐size MSNs in tumor spheroids. Such improvement might be detected by MTC‐chip because the TME‐system on chip could partly replicate the in vivo microenvironment. We hope that our system would promote the development of microfluidic‐based methods for better understanding of the transfer process of NPs.

## 4 Experimental Section

*Device Design and Fabrication*: Microstructure patterns were first designed in AutoCAD software and fabricated using standard photolithography and molding processes, as shown in Figure S1 in the Supporting Information. The printed out as 10 µm resolution chrome masks were produced by Jixian optoelectronic Inc. (Shenzhen, China). Briefly, the pattern of microchannel layer was made from a 20 µm thick SU8‐3025 photoresist patterned on a silicon wafer using photolithography, and the pattern of microwell array layer was made from a 150 µm thick SU8‐2075 photoresist. Next, the silicon wafers (4 in.) were crosslinked via UV light for 6.5 s for microchannel layer and 30 s for microwell array pattern. Subsequently, the designed pattern was developed and cleaned with isopropyl alcohol and nitrogen gas. The silicon masters were baked at 150 °C for 30 min and then treated with the trimethylchlorosilane (Sigma‐Aldrich) via vapor reaction for 4 h.

Polydimethylsiloxane (PDMS RTV615) was obtained from Momentive Performance Materials (Waterford, NY). Next, the structure on the silicon wafer was used to fabricate the PDMS layer (5:1 for microchannel layer and 10:1 for multiple tumor array layer). The mold and PDMS layers were baked at 80 °C for 2 h, and the cured PDMS was cut and removed from the mold. The holes for the inlets and outlets were punched using needle sizes that were compatible with the size of the fluid input/output pins. The PDMS layers were then cleaned by briefly rinsing with isopropyl alcohol and deionized water and dried with nitrogen gas. After treatment with oxygen plasma, the multiple tumor array layer was bonded immediately to a glass slide. Then the multiple tumor array layer was casted a solution of Pluronic F‐127 (1%) on the surface of microwells at room temperature (RT) for 2 h. Next both of the microchannel layer and the multiple tumor array layer were treated with oxygen plasma and trimmed, cleaned, and aligned. Finally, the bonded device was baked for 2 h at 80 °C.

*Cell Culture*: MCF‐7 cell lines were obtained from the American Type Culture Collection (ATCC; Manassas, VA). Cells were grown in Dulbecco's modified Eagle medium (DMEM, Gibico), which was supplemented with 10% vol/vol fetal bovine serum (FBS, Gibico) and 1% penicillin‐streptomycin, at 37 C in a humidified atmosphere of 5% CO_2_.

*3D‐Spheroid Formation and Characterization of Spheroids*: Cells were harvested by treatment with 0.25% trypsin‐ethylenediaminetetraacetic acid (Gibico). The cells were then resuspended in DMEM with 5% Matrigel (Corning Matrigel, Cat#: 356234) for sample injection. 20 µL of 5 × 10^6^ cells mL^−1^ cell suspension was injected into the microchannel using a syringe pump at a 2 µL min^−1^ flow rate for cell loading. The microfluidic device was sterilized by ozone before cell loading. Then the chip was briefly rinsed with 75% ethanol, sterilized water, and phosphate‐buffered saline (PBS) before cell loading. Most of cells flow into the microwell via the gravity force during cell loading. Then the device was incubated for 7 days at 37 °C in a humidified 5% CO_2_ atmosphere. During the spheroids formation, culture medium was replaced every 2 days and the growth and aggregation of the tumor cells were measured under an inverted light microscope (Olympus, X81). After 7 days of incubation, the formed spheroids on MTC‐chip were evaluated using a Calcein‐AM/PI Double Stain Kit (Cat#: 40747ES76, Yeasen, China). For quantitative analysis of the viability, cell survival in 3D tumor was calculated as viability (%) = *A*_s_/(*A*_s_ + *A*_d_) × 100, where *A*_s_ is the area (pixels) of live cells in 3D tumor and *A*_d_ is the area (pixels) of dead cells in 3D tumor.

*Cell Staining*: 3D tumors within device were fixed in 4% paraformaldehyde in PBS for 15 min followed by treated with 0.3% Triton X‐100 in PBS for another 15 min at RT. For immunofluorescence analysis, the fixed samples were then loaded with antibody for E‐CAD (clone 67A4 Biolegend, USA) for 1.5 h at 4 °C, and then the samples were washed with PBS for 10 min at 10 µL min^−1^ to remove excess antibodies. They were viewed under an Olympus, X81 fluorescent microscope. For 3D morphological analysis, the fixed sample was incubated with TRITC Phalloidin (100 × 10^−9^ m, Cat#:40735ES75, Yeasen, China) (F‐actin staining) and 4′,6‐diamidino‐2‐phenylindole (DAPI, 2 ng mL^−1^, Cat#: 40727ES10, Yeasen, China) (nuclei staining) in the dark for 15 min. Imaging was taken by an Olympus, X81 fluorescent microscope. For ECM components determination, spheroids were collected by peeling off the top layer of the chip after spheroids formation, fixed, permeabilized in 5% wt/vol bovine serum albumin /0.1% vol/vol Triton‐X100, and stained with Anti‐Collagen IV antibodies (ab6586, Abcam) and DAPI. Imaging was taken by an Olympus, FV1200 confocal laser scanning microscope.

*Synthesis of MSNs*: The small‐sized MSNs (45 nm size) were synthesized via the slightly modified method reported by Meng et al.^38^ Briefly, cetyltrimethylammonium chloride (10.0 g, Aldrich) was dissolved in 200 mL of water in 500 mL conical flask, followed by stirring at 350 rpm for 15 min at 85 °C. 7.5 mL of tetraethyl orthosilicate (TEOS, Aldrich) was added slowly for 8 min at 85 °C under 350 rpm stirring rate. Then the mixture was kept stirring for 20 min. Then the resulting product was collected by centrifugation (12 000 rpm for 15 min) and washed by methanol twice. The surfactant was removed by methanol/HCL (500:19 v/v) at 40 °C for 12 h. Finally, the product was washed three times in methanol and water sequentially. The resulting NPs are designated as MSN_45_.

Large‐sized MSNs (90, 150, and 300 nm size) were synthesized via the method reported by Kleitz et al.^27^ Briefly, 1.0 g of cetyltrimethylammonium bromide (Aldrich) and “*x*” g of F127 (*x* = 8, 4, 2 for MSNs with size of 90, 150, and 300 nm, respectively) were mixed in 85 mL of 100% EtOH and 213 mL of 2.9 wt % NH_4_OH solution in 500 mL conical flask. 3.86 mL of TEOS (Aldrich) was added slowly for 4 min by stirring at 500 rpm at RT. Then the mixture was aged for 24 h under static conditions at RT. The surfactant was removed by methanol/HCL (500:19 v/v) at 40 °C for 12 h. Finally, the product was washed three times in methanol and water sequentially. The resulting NPs are designated as MSN*_x_* (*x* = 90, 150, and 300).

Fluorescent NPs were prepared by grafting the fluorescein isothiocyanate as the method reported by Pasqua et al.^39^ For this, (3‐aminopropyl)triethoxysilane (APTES, 1%, molar ratio APTES/TEOS) was added to the reaction after the addition of TEOS for MSN*_x_* (*x* = 45, 90, 150, and 300), respectively. Then, a solution of fluorescein isothiocyanate (FITC, Aldrich) (4 mg mL^−1^ in anhydrous dimethyl sulfoxide) was added to the suspension of MSN*_x_* (100 mg in 10 mL of methanol). The mixture was kept under gentle stirring at room temperature for 24 h, in the dark. The suspension was centrifuged and the supernatant was discarded. The solid residue was washed three times with methanol and twice with water sequentially and freeze‐dried at last. The resulting fluorescent material was named as MSN*_x_*‐FITC.

The hydrodynamic diameter of the MSN*_x_*‐FITC was determined by dynamic light scattering using a Zetasizer ZS90 (Malvern Instruments Ltd., USA). And the morphological observation was performed under transmission electron microscopy (JEM‐1200EX) under an acceleration voltage of 200 kV.

*Loading of the MSNs*: NPs penetration was investigated after most of multicellular spheroids reached the size around 150 µm. In this study, two independent syringe pumps were used to load different solutions into the MTC‐chip. Particularly, pump A was used to load media which contained NPs while pump B was used to load blank media, both two flow were mixed in the special hand‐made inlet and injected demanded amount of NPs into the microfluidic device by adjusting the flow rate of two syringe pumps separately. The linear velocity of the fluid in the microchannel on chip was accessed based on the Microfluidic Calculator, which was presented in https://www.dolomite-microfluidics.com/support/microfluidic-calculator/. For evaluating the effect of the route of administration on MSN_90_ penetration, the sampling procedure was set as following: 1) for continuous administration (IV infusion), 3.75 ng µL^−1^ of MSN_90_ was loaded at the total flow speed of 15 µL min^−1^ for 240 min, the dose was 3.75 × 240 × 15 = 13.5 µg; 2) for transient administration (IV bolus), 20 ng µL^−1^ of MSN_90_ was loaded at the total flow speed of 15 µL min^−1^ for 30 min, and then slowly decreased the MSN_90_ concentration to 0 ng µL^−1^ by adjusting pump A and pump B at the total flow speed of 15 µL min^−1^ for another 30 min, the dose was 20 × 30 × 15 + 20 × 30 /2 × 15 = 13.5 µg. For evaluating the effect of the size of MSNs on NPs after a single dose continuous administration NPs delivery, 3.75 ng µL^−1^ of MSN*_x_* was loaded at the total flow speed of 15 µL min^−1^ for 240 min, the dose was 3.75 × 240 × 15 = 13.5 µg. For evaluating the effect of ECM pretreatment of tumor spheroids on MSN300 accumulation, PBS, hyaluronidase (500 µg mL^−1^), Fasudil (2 × 10^−6^ m), and Losartan (50 × 10^−6^ m) were preloaded into the microfluidic device at the flow speed of 15 µL min^−1^ for 10 min per day for 2 days. After the pretreatment, 3.75 ng µL^−1^ of MSN_300_ was loaded at the total flow speed of 15 µL min^−1^ for 240 min, the dose was 3.75 × 240 × 15 = 13.5 µg. After NPs loading, MTC‐chip was washed with PBS for 5 min at 10 µL min^−1^ before observation.

*Whole Chip Imaging*: Imaging was performed using a high‐resolution camera connected to an inverted optical microscope (Olympus, X81) equipped with objectives of 10 × and 20 × magnification. It costed about 10 min for taking the image of the whole chip (10 ×, bright field and FITC). For evaluating the effect of the route of administration on MSN_90_ penetration, time‐lapse images were captured during the whole experiment. For other experiments, images were captured after the samples were washed with PBS.

*Spheroid Image Analysis*: To quantify the fluorescent distribution of MSNs within the spheroid, ImageJ (NIH research services branch) was used. First, the bright field image of each spheroid was used to determine the area of each microwell (red circle, Figure S6, Supporting Information) and quantify the distance from the center to the edge of the spheroid, which was the radius of the tumor spheroid. Thus, the zone of the measurement of fluorescence intensity value was confirmed. Then the surface plot command was used to generate the surface plots of each spheroid. This was followed by the production of total pixel intensity within an obtained spheroid (blue circle, Figure S6, Supporting Information), then the mean intensity was calculated via dividing the total pixel intensity by the area of the spheroid. Meanwhile, the fluorescent intensity in the media within each microwell was assumed as background and was subjected to normalize the average intensity value for each spheroid. Then a customized script edited in ImageJ (NIH research services branch), which can be downloaded from “https://imagej.nih.gov/ij/plugins/radial-profile.html,” was used to quantify the radial distribution of particles from the center to the edge of the spheroid. Next, a circle (yellow circle, Figure S6, Supporting Information) which covered the whole spheroid was drawn to generate the radial distribution, which displayed the pixel intensity at each radial distance from the center to the edge of the spheroid. Distances were then normalized by the spheroid radius at which greatest pixel intensity was found for each spheroid. Within this function, the average radial intensity distribution for each spheroid was obtained based on the data.

*Cellular Uptake of MSNs in 2D Static Incubation*: MCF‐7 cells were seeded in a 96‐well plate in DMEM medium with 10% FBS and cultured in a humidified atmosphere containing 5% CO_2_ at 37 °C for 24 h before MSNs incubation. For cell uptake evaluation, MSNs were dispersed in free DMEM medium (0.5 mg mL^−1^), then the cells were further incubated at 37 °C for another 4 h. After removing the medium and washing with PBS, the cells were observed under a fluorescent microscopy (Olympus, X81). The relative fluorescence intensity was calculated by normalizing each cell's intensity by its area.

*Cellular Uptake of MSNs in 3D Static Incubation*: MCF‐7 cells were seeded and cultured on ultra‐low attachment plates for spheroid formation. On day 7 of the culture, the spheroids were replaced with media containing MSNs (0.5 mg mL^−1^) and were left to incubate for 4 h. After removing the medium and washing with PBS, the cells were observed under a fluorescent microscopy (Olympus, X81). The relative fluorescence intensity was calculated by normalizing each spheroid's intensity by its area.

*Human Liver Organoids (PLOs) Chip Culture*: Human tissue and specimens were obtained by surgical resection from patients of primary liver cancer. Ethical approval for this study was obtained from Ethics Committee of the First Affiliated Hospital of Sun Yat‐sen University (Approval number/ [2018] 43). Written informed consents were all obtained from patients. The tumor surgical resections were mechanically disintegrated into small pieces (0.5–1 mm^3^) and incubated with the digestion solution, which contained 2.0 mg mL^−1^ collagenase IV (Sigma‐Aldrich), 0.1 mg mL^−1^ DNase (Sigma), and 10 × 10^−6^ m Y‐27632 (Sigma) for 30 min. Then the digested cell suspension was filtered through 100 µm nylon net and centrifuged for 5 min at 300 *g*. Cells were resuspended in the 50% reduced growth factor BME2 (Basement Membrane Extract, Type 2; R&D) (dissolved in expansion medium) with the cell density at 500 000 mL^−1^. The expansion medium is advanced DMEM/F12 (Gibco) supplemented with 1% penicillin/streptomycin (Gibco), 1% glutamax (Sigma), 10 × 10^−3^ m 4‐(2‐hydroxyethyl)‐1‐piperazineethanesulfonic acid (Sigma), 1:50 B27 supplement (Gibco), 1:100 N_2_ supplement (Gibco), 1.25 × 10^−3^ m n‐acetyl‐L‐cysteine (Sigma), 10% Rspo‐1 conditioned medium (homemade), 30% Wnt3a conditioned medium (homemade), 10 × 10^−3^ m nicotinamide (Sigma), 10 × 10^−9^ m recombinant human [Leu15]‐Gastrin I (Sigma), 50 ng mL^−1^ recombinant human EGF (PeproTech), 100 ng mL^−1^ recombinant human FGF10 (PeproTech), 25 ng mL^−1^ recombinant human HGF (PeproTech), 5 × 10^−6^ m A8301 (Tocris), 25 ng mL^−1^ Noggin (PeproTech), and 10 × 10^−6^ m Y27632 (Tocris). Then the cells were loading into the modified chip (the height of microchannels was adjusted to 50 µm) at the flow rate of 4 µL min^−1^ for 5 min. The chip was incubated at 37 °C for 20 min until the medium was solidified. The organoid expansion medium was changed every 3 days by medium flushing. After 7 days culture, the organoids were imaged by an Olympus, X81 fluorescent microscope for the morphological analysis. Then the top layer of the chip was peeled off. The organoids were collected, fixed, permeabilized, and incubated with anti‐AFP antibody (ab46799, abcam) and DAPI. Imaging was taken by an Olympus, FV1200 confocal laser scanning microscope.

*Statistical Analysis*: The whole study was composed of 16 chips. Four chips were used for characterization of spheroids, including live/dead staining, 3D morphological analysis, immunofluorescence analysis, and the characterization of tumor spheroids growth. Three chips were used for the study of the route of administration on MSNs penetration. Four chips were used for the study of the effect of the size of MSNs on NPs penetration after a single dose continuous administration NPs delivery. And four chips were used for the study of the effect of ECM pretreatment of tumor spheroids on MSN_300_ accumulation. One chip was used for the organoids culture. All obtained data were expressed as the mean ± standard deviation. For the NPs cellular uptake analysis, significance was assessed by Student's *t* test, and *p*‐values < 0.05 were considered to be statistically significant.

## Conflict of Interest

The authors declare no conflict of interest.

## Supporting information

Supplementary
