# Supplementary material for: A Dynamic 3D Tumor Spheroid Chip Enables More Accurate Nanomedicine Uptake Evaluation
Source: Adv Sci (Weinh). 2019 Oct 4;6(22):1901462. doi: 10.1002/advs.201901462 (PMC6864993; doi:10.1002/advs.201901462)
Supplement: Supplementary file 1 — Supplementary [file ADVS-6-1901462-s001.pdf]

Copyright WILEY-VCH Verlag GmbH & Co. KGaA, 69469 Weinheim, Germany, 2019.

ADVANCED SCIENCE
Open Access

Supporting Information

for *Adv. Sci.,* DOI: 10.1002/advs.201901462

A Dynamic 3D Tumor Spheroid Chip Enables More Accurate Nanomedicine Uptake Evaluation

*Jialang Zhuang, Jie Zhang, Minhao Wu, and Yuanqing Zhang**

Supplementary information

A Dynamic 3D Tumor Spheroid Chip Enables More Accurate Nanomedicine Uptake Evaluation

Jialang Zhuang[1], Jie zhang[1], Minhao Wu[2], and Yuanqing Zhang[1]*

[1]Guangdong Key Laboratory of Chiral Molecule and Drug Discovery, School of Pharmaceutical Sciences, Sun Yat-sen University, Guangzhou 510006, P. R. China;

[2]Department of Immunology, Zhongshan School of Medicine, Sun Yat-sen University, 74 Zhongshan 2nd Road, Guangzhou 510080, P. R. China;

Email：zhangyq65@mail.sysu.edu.cn

includes:
Supplementary Figures. S1-12
Supplementary Table S1

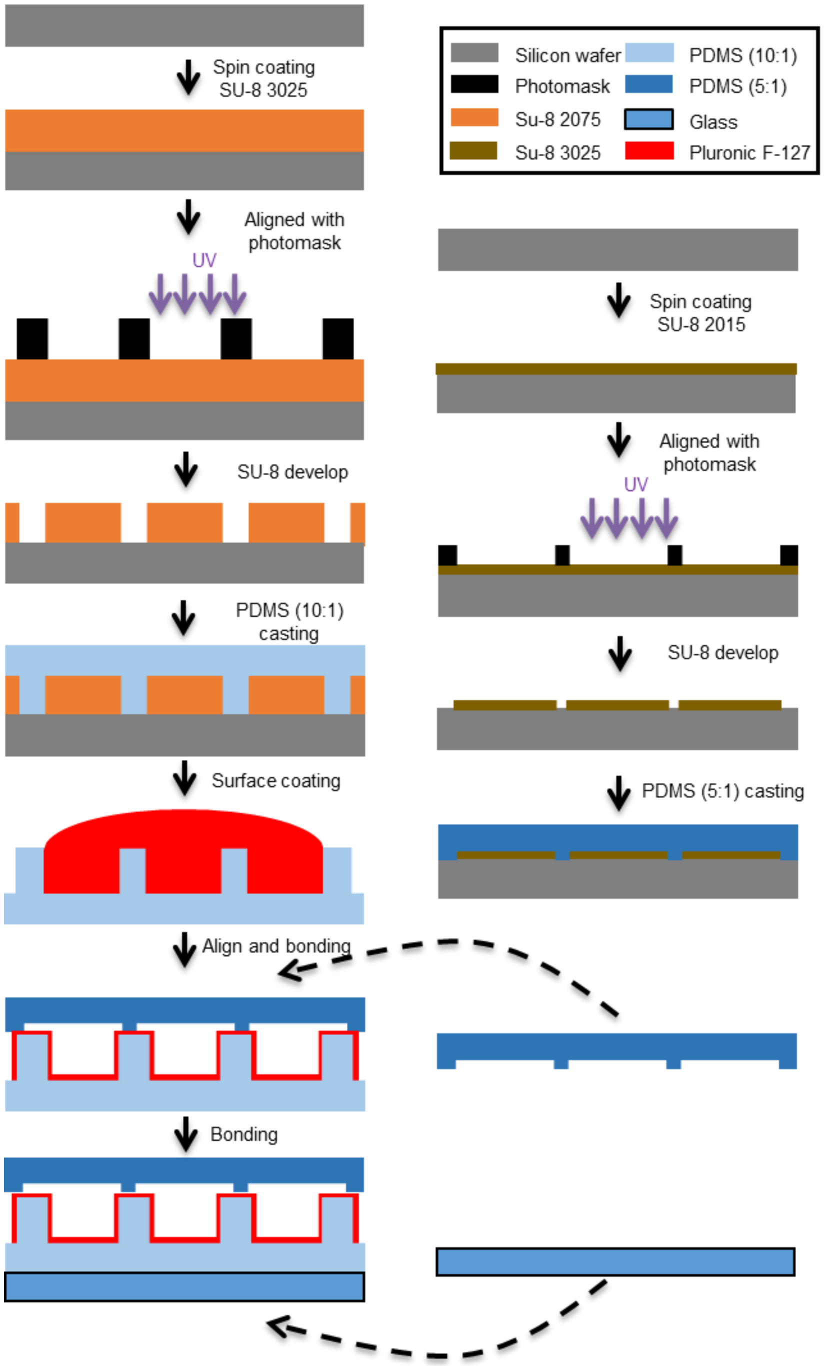

Figure S1. Schematic representation of the fabrication process.

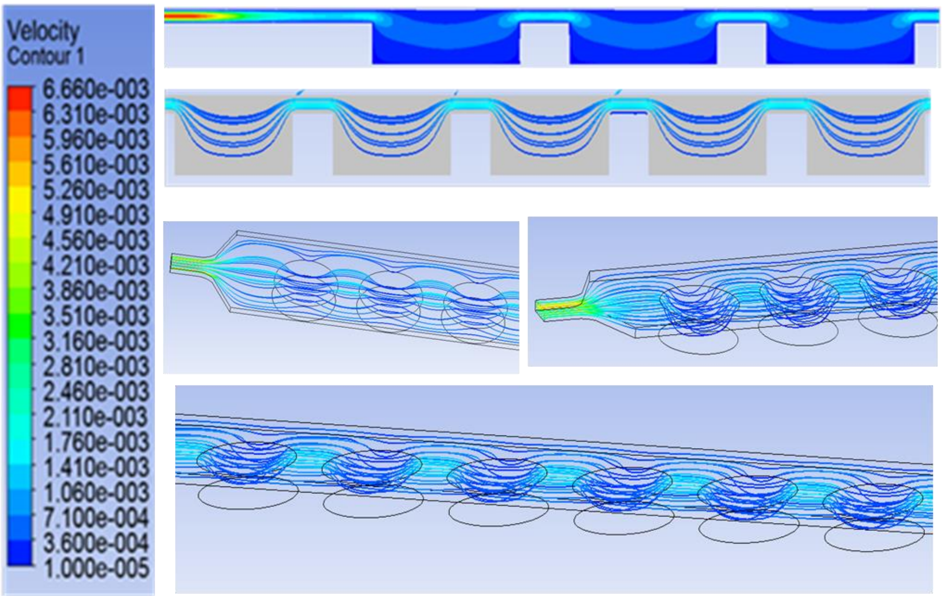

Figure S2. Simulation of microfluid system was conducted by FLUENT software. Fluid dynamic analysis showed that directed fluid flow into the microchannel prefer to filling with the microwells, which can drive cells fall into the microwells.

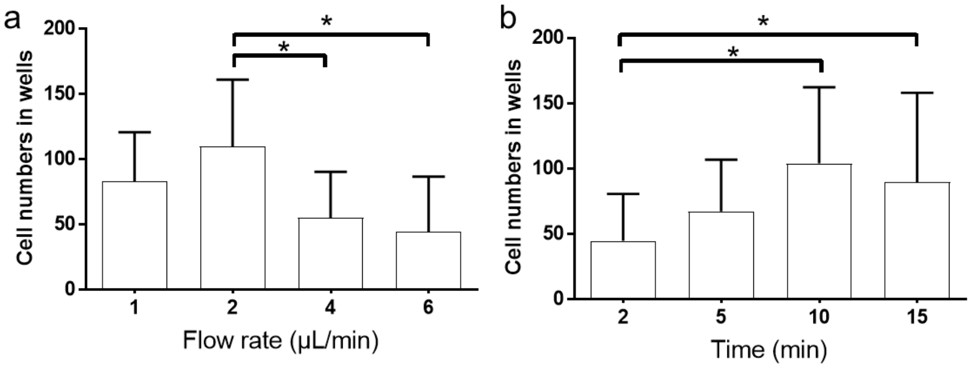

Figure S3. Optimization of microfluidic device for cell loading.

Cell numbers in microwells after cell loading under various flow rate a) and loading time b).

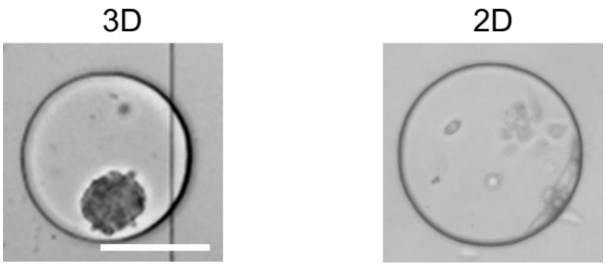

Figure S4. Representative images of MCF-7 cells in the microwells. The cells were 3D cultured and 2D cultured on chip, scale bar, 200 μm.

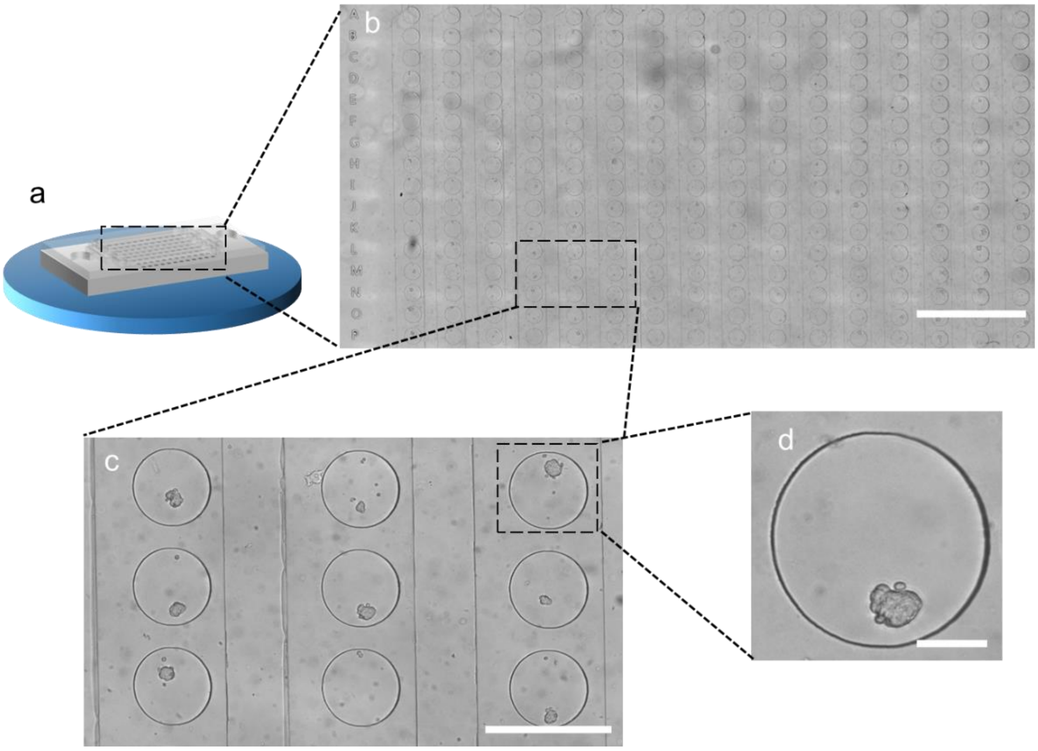

Figure S5. Optical micrographs of 3D cell culture of MCF-7 on chip for 1 day.

a) Schematic representation of the microfluidic chip. b) the whole view of the micro-device after cell culture for 1 day, there are 16 microchannels on chip while each microchannel contains 16 microwells for cell culture, scale bar, 2 mm, c) most of the 3D tumor were settled in the microwells, scale bar, 500 μm, and the d) Bright-field image of single MCF-7 spheroid within the microwells, scale bar, 100 μm.

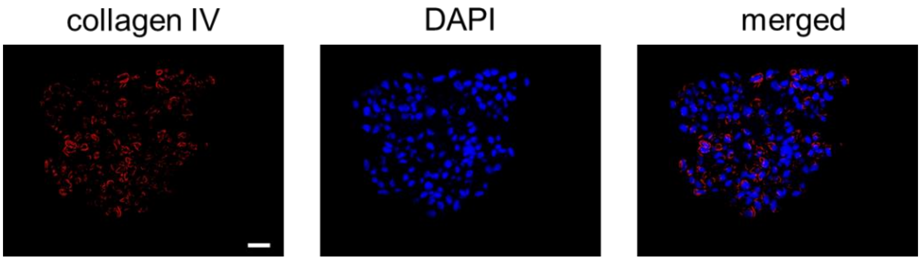

Figure S6. Visualization of collagen IV in MCF-7 spheroids developed by MTC-chip.

The spheroids were fixed and stained with anti-collagen IV antibody and DAPI after collection from the device, scale bar, 20 μm.

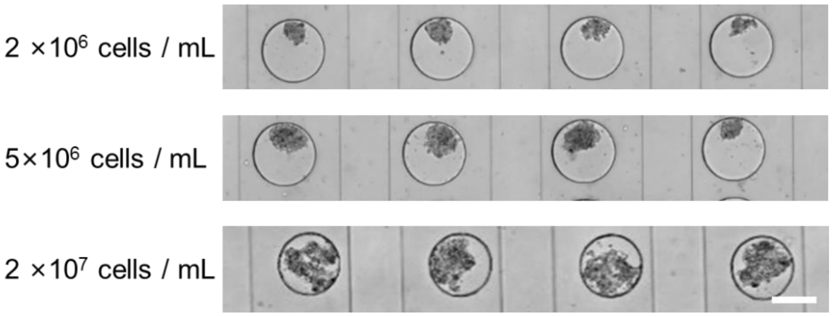

Figure S7. The effect of cell density on MCF-7 3D culture on chip. 20 μL of 2 $\times 10^6$, 5 $\times 10^6$ and 2 $\times 10^7$ cells / mL cell suspension were injected into the microchannel using a syringe pump at a 2 μL/min flow rate for cell loading. The chips were incubated in a humidified atmosphere containing 5% CO_2 at 37°C for 7 day before imaging with a microscope, scale bar, 200 μm.

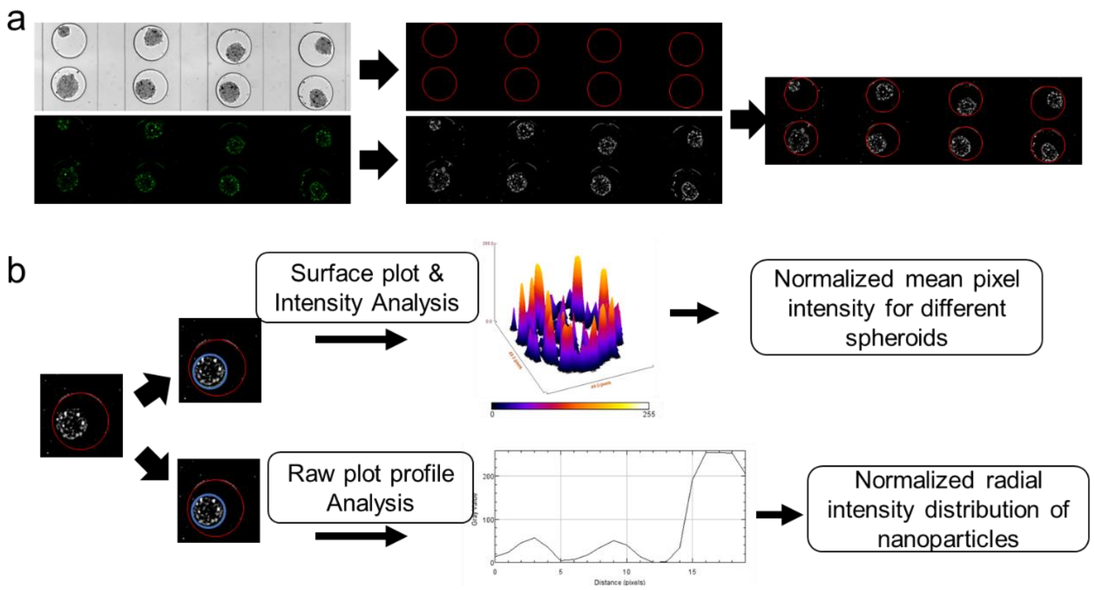

Figure S8. Procedure of quantification of fluorescent intensity of MSNs within MCF-7 spheroids.

a) Workflow of the selection of the analysis area on chip by the bright-filed images and the fluorescent images, the red circles indicate the edges of the microwells. b) Workflow of the production of surface plots, the mean pixel intensity and normalized fluorescent intensity distribution for different spheroids. The fluorescent image of one MCF-7 spheroid was boxed off (red circle) and was subjected to surface plot command and radial distribution function command by imagej. The blue circle indicates the area of the given spheroid which was

internalized with MSNs.

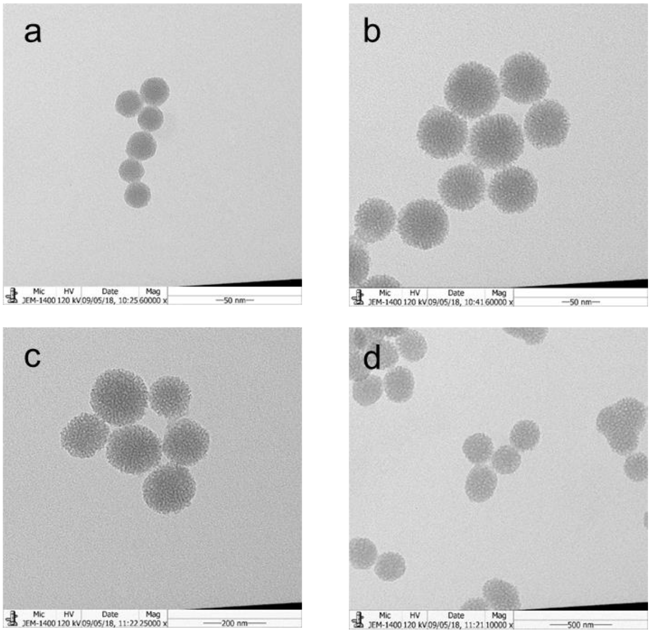

Figure S9. TEM images of MSNs with controllable diameters.

TEM images of spherical MSNs with 45 nm a), 90 nm b), 150 nm c) and 300 nm d).

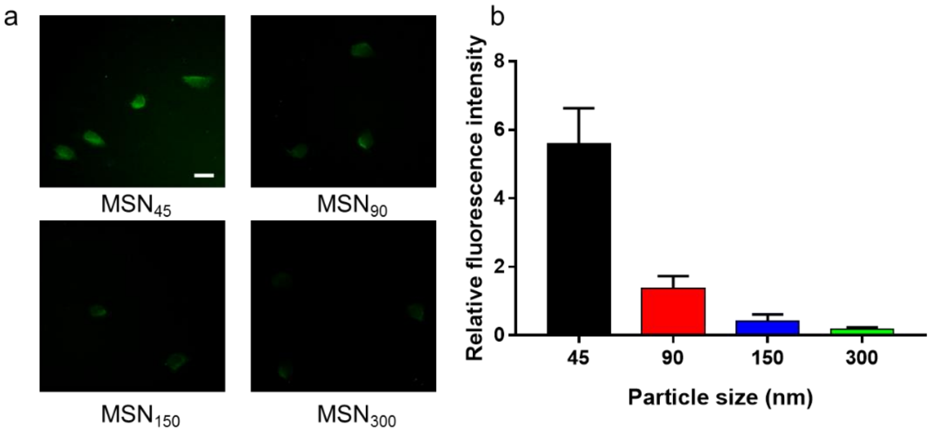

Figure S10. Cellular uptake of different size of MSNs by 2D monolayer culture system.

Fluorescence images a) and the relative fluorescence intensity per cell b) of MCF-7 cells treated with different size of MSNs after 4 hour, scale bar, 10 μm.

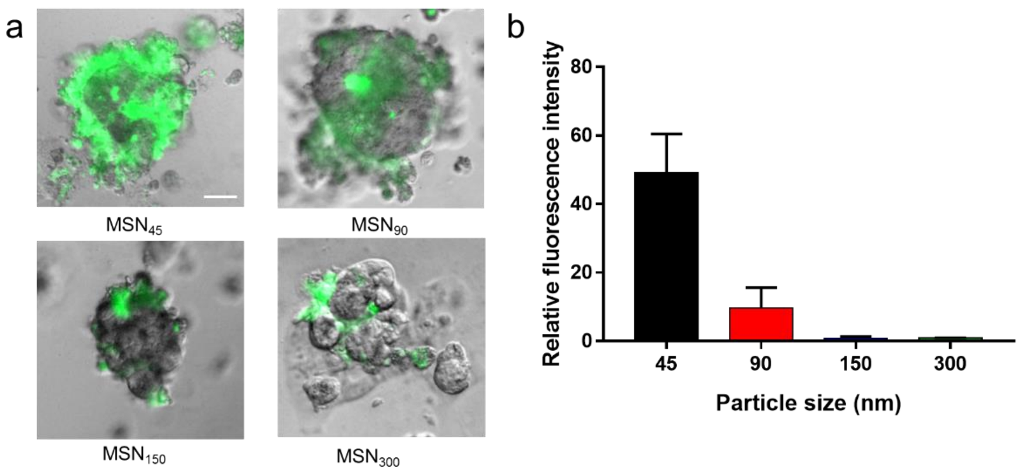

Figure S11. penetration of MSNs with different size into spheroids. Fluorescence images (a) and the relative fluorescence intensity per spheriod (b) of MCF-7 cells treated with different size of MSNs after 4 hour, scale bar, 20 μm.

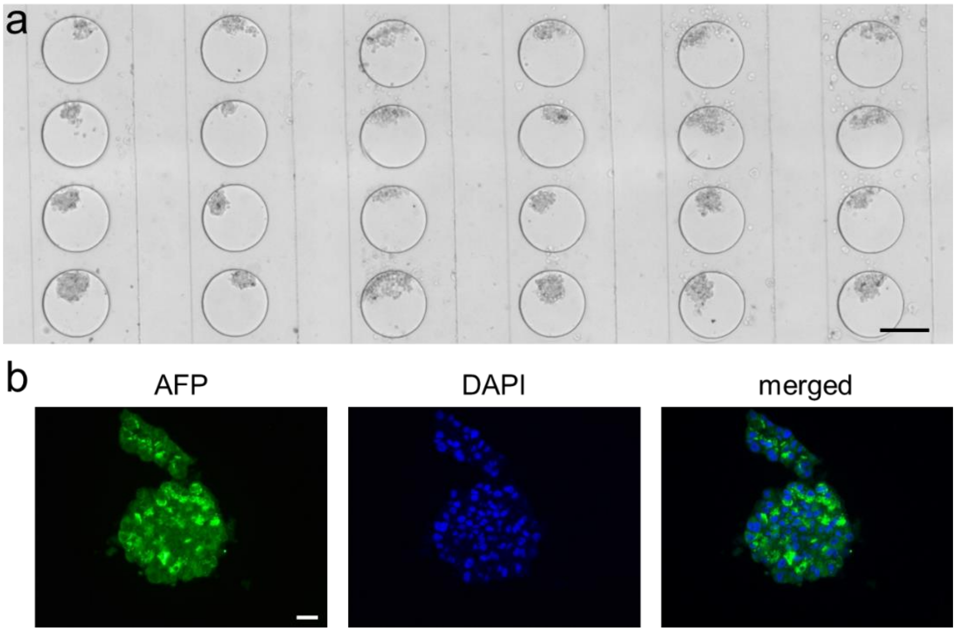

Figure S12. Human primary liver cancer-derived organoids culture in MTC-chip. Bright-field image (a) and confocal immunofuorescence micrographs (b) of organoids cultured on chip for 7 days, scale bar, 200 μm (a) and 20 μm (b). Heterogeneous morphologies of organoids were observed on chip. The Alpha-fetoprotein (AFP) was detected in the cultured organoids.

Table S1. Particle size and polydispersity index (PDI) of MSNx with different diameters

MSNx	Size (nm)	PDI

MSN_{45}	58.6 ± 2.8	0.022 ± 0.020
MSN_{90}	87.5 ± 9.3	0.045 ± 0.012
MSN_{150}	169.6 ± 10.5	0.058 ± 0.001
MSN_{300}	315.9 ± 17.6	0.162 ± 0.013
